# Exploring Clinical Risk Factors for Breast Cancer Among American Indian Women

**DOI:** 10.3389/fpubh.2022.840280

**Published:** 2022-06-17

**Authors:** Melanie Nadeau, Lyle G. Best, Marilyn G. Klug, Kathryn Wise

**Affiliations:** ^1^Department of Indigenous Health, School of Medicine and Health Sciences, University of North Dakota, Grand Forks, ND, United States; ^2^Turtle Mountain Community College, Belcourt, ND, United States; ^3^Department of Population Health, School of Medicine and Health Sciences, University of North Dakota, Grand Forks, ND, United States

**Keywords:** American Indian, breast cancer, epidemiology, risk factors, health disparities

## Abstract

**Objective:**

Very little is known about the breast cancer risk profile among American Indian women. Previous research shows that the proportion of American Indian/Alaska Native women with baseline characteristics (commonly known breast cancer risk factors) differs from other ethnicities. This retrospective case control study was designed to the explore the association of these factors among American Indian women with and without breast cancer.

**Methods:**

Cases and controls were retrospectively selected from the medical records of American Indian women who obtained their health care from Quentin N. Burdick Memorial Health Care Facility (Indian Health Service) in Belcourt, ND. For each woman with breast cancer (*n* = 141), two controls were selected when possible (*n* = 278). Risk factors examined included woman's age, age at first live birth, age of menarche, the number of previous benign breast biopsies, the total number of first-degree relatives with breast cancer, body mass index and parity. Odds ratios and 95% confidence intervals were calculated using logistic regression.

**Results:**

Many of the associations found among American Indian women who obtained their health care from Quentin N. Burdick Memorial Health Care Facility (Indian Health Service) in Belcourt, ND, between risk factors commonly identified in other populations and breast cancer were weakly positive. Nulliparity was the only risk factor to consistently show a positive significant association (OR = 2.87, 95% CI 1.16–0.7.12).

**Conclusion:**

Disparities in breast cancer incidence, mortality and screening among Northern Plains American Indian emphasize the need to better understand the risk factors associated with breast cancer in this population. Based on the results of this study, the value of current risk prediction models in American Indian communities is uncertain and clinicians should be cautious in using these models to inform American Indian patients of their risk for breast cancer.

## Background

American Indian women are significantly impacted by breast cancer incidence and mortality. According to the American Indian Cancer Foundation, 1 in 8 American Indian women will get breast cancer in their lifetime (1). Nationally, the incidence rates for breast cancer among American Indian/Alaska Native females from 2014 to 2019 were 110.5 per 100,000 compared to 126.9 per 100,000 for all races combined (2). During this same period, the mortality rates for breast cancer among American Indian/Alaska Native females were 17.8 per 100,000 compared to 19.9 per 100,000 for all races combined (2). Compared to White people, the risk of death for cancer after adjusting for sex, age and stage at diagnosis was 51% higher in American Indian/Alaska Native people (2).

Very little is known about the breast cancer risk profile among American Indian women. In 2005, Chlebowski et al. examined data from the Woman's Health Initiative for 156,570 postmenopausal women recruited from 40 health facilities across the United States, ages 50 to 70 years with American Indians/Alaska Natives representing 0.4% (*n* = 696) (3). The proportion of women with breast cancer risk factors by race/ethnicity was reported (3). Results showed that the proportion of women with known risk factors including age, age at first live birth, age at onset of menstruation, the number of previous benign breast biopsies, the total number of first-degree relatives with breast cancer, BMI, and parity differs between other ethnicities and American Indians/Alaska Natives. According to the study, American Indian women had a higher percentage of first-degree relatives with breast cancer, a higher percentage of individuals whose age at first occurrence of menstruation was older than age 14, a lower percentage who had never given birth and generally a higher percentage of individuals with a BMI ≥ 30 compared to other ethnicities (3). However, the small number of American Indians/Alaska Natives included (*n* = 696, 11 of whom developed invasive breast cancer), limited the author's ability to make conclusions about the associations of these factors with breast cancer (3).

Historically, breast cancer risk factor data has been used to inform the creation of breast cancer risk assessment tools. A breast cancer risk calculator is a mathematical model that is commonly used by physicians to determine a woman's risk of breast cancer. Physicians also use information from these models to inform their patients about what risk factors are increasing their risk of breast cancer. Some of the more commonly known calculators are Gail, and Rosner and Colditz (4). Other prediction models have also been constructed by combining risk factors from these commonly known models (4). Most models have been studied in White populations and some models have been studied in mixed populations with White being the majority (4). Only a few models have been developed using data from minority populations, Asians and African Americans (4). These models did not perform as well as other Gail models (4, 5). A breast cancer risk calculator using data from American Indian women has not been developed.

This study explored risk factors included in the Gail model as well as BMI and parity for an American Indian population in North Dakota. Given that the model was adjusted for the same risk factors in other populations, it was hypothesized that the risk factors would be the same in the American Indian population; however, the magnitude of the associations would likely differ.

## Research Methods

### Study Design

This study was a quasi-case-control study design and was undertaken to assess the association between potential risk factors and breast cancer among a population of American Indian women who obtained their health care from Quentin N. Burdick Memorial Health Care Facility (Indian Health Service) in Belcourt, ND. The study was approved by the Turtle Mountain Band of Chippewa Indians Research Review Board, Great Plains Institutional Review Board (IRB) and the University of Minnesota IRB. The original study was completed while lead author (Nadeau) was at the University of Minnesota, hence the IRB was included from that location. A resolution in support of the study was also passed by the Turtle Mountain Band of Chippewa in Belcourt, ND.

An electronic database, the Resource and Patient Management System, at the Indian Health Service was queried using ICD-9 code 174 to retrospectively identify patients with a breast cancer diagnosis (cases) from May 1990 through January 29th, 2016. Case and control ethnicity was defined as American Indian by virtue of their eligibility for care within the Indian Health Service and was subsequently verified in each medical record. A total of 170 patients with a possible breast cancer diagnosis were identified. A pathological diagnosis was confirmed for 141 (82.94%) patients. The remaining 29 were eliminated for the following reasons: unable to find risk factor data (*n* = 1), unable to identify controls (*n* = 2), unable to retrieve chart from archives (*n* = 4), no previous mammogram (*n* = 9), and unable to confirm a pathological diagnosis for breast cancer (*n* = 13).

For each case, we randomly selected, at most, two controls from women who had a mammogram at the facility and had not had a breast cancer diagnosis. Controls were matched so their age was within 5 years of the cases. The two controls selected also had to have mammograms that were the closest to the date (pre or post) of the diagnostic mammogram of the case. Due to matching restrictions, there were four cases where we could only find one control. This resulted in a total of 141 cases and 278 controls with an age range of 29 to 88 years of age.

### Data Collection

Since 1986, the Quentin N. Burdick Healthcare Facility asked every woman undergoing a mammogram to complete a brief questionnaire that included the following questions about risk for breast cancer: date of birth, age at first pregnancy, menstrual history – age at onset, ever had breast surgery (if yes, the patient is asked to specify mastectomy, biopsy, aspiration, other, right and/or left, and date), and if any blood relative had breast cancer (if yes, the patient is asked to specify mother, sister, grandmother, aunt, age of each relative identified and whether the relative was maternal or paternal). These questionnaires, kept in the Radiology department, served as the primary source of data for the study.

Each study participant was assigned a unique study ID which was linked to their chart number on a list that was kept in a secure area at the Indian Health Service facility. Data was recorded for risk factors included in the study. Date of previous benign breast biopsy, date of mastectomy and if the patient had an active radiology, electronic, and hard chart on file were also recorded. It was also noted whether review was completed for the radiology, electronic and medical chart files (see Table 1A in Appendix). Personal computers are not allowed in secured areas at the facility, so all data was entered into an Excel spreadsheet off site. No identifying data was collected. When possible, the first BMI documented before and after mammogram and number of live births was collected from the electronic medical record. Otherwise, this information along with age at first live birth was collected from the chart.

### Models and Variable Categorization

Two approaches were taken to examine risk factors for breast cancer. In Phase I, risk factors and the categorization used in the Gail model were included. In Phase II, risk factors were categorized according to their distribution in the population of American Indian women.

The following Gail model categorization was used for Phase I of the analysis: age at screening (<50, ≥50), age at first live birth (<20, 20–24, 25–29, ≥30, never given birth), age of onset of menstruation (<12, 12–13, ≥ 14 years), the number of previous benign breast biopsies (0, 1, ≥ 2), the number of first-degree relatives with breast cancer (0, 1, ≥2), BMI (<25, 25–30, >30), number of live births (1, 2, 3, 4, ≥5, never given birth).

For Phase II of the analysis, the distribution of the data was assessed, and new adjusted categories were proposed. Compared to the general population, American Indian women in this study were screened at an older age and were younger at age of first live birth (Table 1A in Appendix). They also had fewer previous benign breast biopsies and first-degree relatives with breast cancer, a higher BMI and more live births compared to the general population.

After review, the adjusted categories were finalized for the study population and the data was re-analyzed. The adjusted categories used for Phase II of the analysis were as follows: age at screening (<56, ≥56), age at first live birth (<18, 18–19, 20–21, ≥22, never given birth), age of onset of menstruation (<12, 12, 13, ≥14 years), the number of previous benign breast biopsies (0, ≥ 1), the number of first-degree relatives with breast cancer (0, ≥ 1), BMI (<25, 25–29.99, 30–32.49, 32.50–34.99, ≥35), number of live births (1–2, 3–4, ≥5, never given birth).

### Statistical Analyses

The following statistical models were computed for both Phase I (original categories) and Phase II (adjusted categories) of the analyses, (1) logistic regression using the Gail model (woman's age, age at first live birth, age of onset of menstruation, the number of previous benign breast biopsies, and the total number of first-degree relatives with breast cancer), (2) logistic regression of the Gail model with BMI added, and (3) logistic regression of the Gail model with both BMI and parity added (first live birth was removed to avoid multicollinearity complications with parity). Odds ratios (OR) along with 95% confidence intervals (CIs) were reported. The c-statistic was used to test the model as a whole.

## Results

Medical record review occurred retrospectively for 141 cases and 278 controls matched by age of women and date of breast cancer screening at Quentin N. Burdick Memorial Health Care Facility (Indian Health Service) in Belcourt, ND. Five variables from the Gail model for predicting risk of breast cancer were used in this study (age at screening, age at first live birth, age at onset of menstruation, number of previous benign breast biopsies, total number of first-degree relatives with breast cancer). Table 2A in Appendix shows the distribution of these variables' categories for cases and controls. There was little difference in these variables between cases and controls for this population.

When multivariate analysis, restricted to the four risk factors included in the Gail model, was conducted, women who had never given birth were over twice as likely to have had breast cancer compared to women whose age at first live birth was before age 20 (OR = 2.54, CI 1.07 to 6.02; Table 1). Women who had an onset of menstruation of 12 to 13 years old also had increased risk of breast cancer relative to those who began after age 13 (OR = 1.62, CI 1.01 to 2.61). No other variables from the Gail model (age, previous biopsies, and relatives with breast cancer) significantly increased the risk of breast cancer.

**Table 1:** Logistic regressions using risk factors from Gail model with additions of BMI and parity.

	**Gail**	**Gail + BMI**	**Gail + Parity**
	**OR (95% C)**	**OR (95% C)**	**OR (95% C)**
Age at screening			
<50 (ref) ≥50	1.09 (0.69–1.72)	1.10 (0.69–1.74)	1.14 (0.70–1.85)
Age at first live birth			
<20 (ref) 20 to 24 25 to 29 ≥30 Nulliparous (never given birth)	0.84 (0.53–1.31) 0.72 (0.25–2.12) 0.68 (0.17–2.69) **2.54 (1.07–6.02)**	0.83 (0.53–1.31) 0.72 (0.25–2.10) 0.67 (0.17–2.64) **2.52 (1.06–6.00)**	
Age at onset of menstruation			
≥14 (ref) 12 to 13 <12	**1.62 (1.01–2.61)** 1.14 (0.61–2.15)	**1.67 (1.03–2.70)** 1.18 (0.62–2.24)	**1.66 (1.03–2.70)** 1.21 (0.64–2.29)
Number of previous benign breast biopsies			
0 (ref) 1 ≥2	0.99 (0.57–1.71) 1.71 (0.50–5.91)	0.99 (0.57–1.71) 1.68 (0.49–5.79)	0.99 (0.57–1.72) 1.66 (0.49–5.73)
Total number of first-degree relatives with breast cancer			
0 (ref) 1 ≥2	1.26 (0.75–2.10) 1.53 (0.51–4.51)	1.26 (0.75–2.10) 1.54 (0.52–4.57)	1.25 (0.75–2.09) 1.38 (0.47–4.07)
BMI, kg/m^2^			
<25 (ref) 25 to 30 >30		1.06 (0.51–2.19) 0.88 (0.44–1.78)	1.04 (0.50–2.16) 0.87 (0.43–1.75)
Parity (# of live births)			
≥5 (ref) 4 3 2 1 Nulliparous (never given birth)			0.97 (0.49–1.89) 1.04 (0.58–1.87) 1.46 (0.78–2.72) 1.32 (0.53–3.29) **3.02 (1.23–7.40)**

*When parity was added to the model, age at first live birth, highly collinear, was removed. The bold values indicate statistically significant results*.

When BMI was included with the Gail model, the change in odds ratios for all variables was minor (Table 1). Women who had never given birth and those who had an onset of menstruation of 12 or 13 was still significant. Age at first live birth was then replaced with parity (number of live births). Again, there was little change in the odds ratio for age at menstruation. Women who had never given birth had a threefold increase in risk of breast cancer (OR = 3.02, CI 1.23 to 7.40) compared to women with five or more children. The c-statistic for all models in the Phase I analysis ranged from 0.59 to 0.61.

For Phase II of the analysis, adjusted categories were proposed based on characteristics of the female American Indian population sampled. The adjusted categories were as follows: age at screening (<56, ≥56), age at first live birth (<18, 18–19, 20–21, ≥22, never given birth), age of onset of menstruation (<12, 12, 13, ≥14 years), the number of previous benign breast biopsies (0, ≥1), the number of first-degree relatives with breast cancer (0, ≥ 1), BMI (<25, 25–29.99, 30–32.49, 32.50–34.99, ≥35), number of live births (1–2, 3–4, ≥5, never given birth). When the risks based on adjusted categories were analyzed, women who had an age at onset of menstruation of 12 was significant (OR = 1.81, CI 1.05 to 3.14; Table 2). This was consistent when BMI was added to the model, and when age at first live birth was replaced with parity. While age at first live birth was not a significant risk of breast cancer, no children compared to having five or more children nearly tripled the risk of breast cancer (OR = 2.87, CI 1.16–7.12). The c-statistic for all models in the Phase I analysis ranged from 0.61 to 0.63. Many of the associations found for American Indian women in North Dakota between risk factors commonly identified in other populations and breast cancer were weakly positive with confidence intervals including the null value.

**Table 2:** Logistic regressions using risk factors from gail model with adjusted categories.

	**Gail**	**Gail + BMI**	**Gail + Parity**
	**OR (95% C)**	**OR (95% C)**	**OR (95% C)**
Age at screening			
<56 ≥56	0.98 (0.64–1.51)	0.98 (0.64–1.52)	1.03 (0.64–1.65)
Age at first live birth			
<18 18 to 19 20 to 21 ≥22 Nulliparous (never given birth)	0.70 (0.39–1.26) 0.63 (0.34–1.15) 0.75 (0.39–1.43) 1.99 (0.79–5.02)	0.70 (0.39–1.26) 0.63 (0.34–1.16) 0.76 (0.40–1.47) 1.97 (0.77–5.00)	
Age at onset of menstruation			
≥14 13 12 <12	1.35 (0.77–2.38) **1.81 (1.05–3.14)** 1.05 (0.55–2.00)	1.40 (0.79–2.47) **1.89 (1.08–3.31)** 1.10 (0.57–2.11)	1.44 (0.82–2.54) **1.93 (1.10–3.38)** 1.20 (0.63–2.27)
Number of previous benign breast biopsies			
0 ≥1	1.08 (0.64–1.81)	1.06 (0.63–1.79)	1.05 (0.62–1.77)
Total number of first-degree relatives with breast cancer			
0 ≥1	1.29 (0.80–2.09)	1.28 (0.79–2.08)	1.27 (0.79–2.06)
BMI, kg/m^2^			
<25 25 to 29.99 30 to 32.49 32.50 to 34.99 ≥35		1.04 (0.49–2.19) 0.97 (0.45–2.10) 1.44 (0.61–3.41) 0.76 (0.35–1.64)	1.01 (0.48–2.13) 0.91 (0.42–1.96) 1.46 (0.62-3.44) 0.73 (0.34–1.58)
Parity (# of live births)			
≥5 3–4 1–2 Nulliparous (never given birth)			1.00 (0.59–1.70) 1.47 (0.81–2.66) **2.87 (1.16–7.12)**

*When parity was added to the model, age at first live birth, highly collinear, was removed. The bold values indicate statistically significant results*.

## Discussions

This study focused on retrospective medical chart review for 141 cases and 278 controls. Aside from nulliparity or having an age at onset of menstruation of 12 or 13, no other associations between the risk factors commonly identified in other populations and breast cancer were found for American Indian women who obtained their health care from Quentin N. Burdick Memorial Health Care Facility (Indian Health Service) in Belcourt, ND. The risk factors that were expected to be positively associated with breast cancer, for the most part, showed a null and/or an inverse relationship. The c-statistic for all models in Phase I and Phase II of the analysis ranged from 0.59 to 0.63. This statistic indicates poor model discrimination which means that the models do not contain variables that are strongly associated with the outcome of breast cancer. If the regression models contained explanatory variables that were strongly associated with the outcome, improved discrimination would be expected (6).

Risk factors for American Indian women in the Great Plains region may differ and/or vary in magnitude compared to those identified in other populations. Additional breast cancer risk factors have been identified over the past two decades but have not been included in any models (4). These risk factors include oral contraceptive use, alcohol use, smoking, diabetes mellitus, menopausal status, and breastfeeding. Several of these recently identified risk factors are likely to occur more frequently in American Indians than other communities suggesting a greater need to include them in predictive risk models.

It is unlikely that a single Breast Cancer Risk Assessment Tool could be developed that would work for all tribes or regions of American Indian women. A total of 574 tribes are federally recognized across the United States, each one differing in their culture, environment, healthcare, and behavioral health, some of them dramatically (7). As a result, the cancer burden can vary significantly from tribe to tribe and these differences could impact the types of risk factors that are present. Though we found little association for standard risk factors in our models, that may not hold true for other tribal areas.

## Limitations

For the study, the availability of medical records data for potential breast cancer variables was limited. Variables previously associated with breast cancer, such as smoking and breast feeding, were unavailable. Further studies may have improved models if they include these variables, reducing variance in the model. Our sample was limited to women who had a mammogram. This may have biased the data if women are only likely to have a mammogram if they have a problem or can easily obtain the procedure. Further, the matching between cases and controls was not exact nor complete. Our conclusions could have been strengthened if the control group was enlarged, or if more precise matching had been available.

Various studies have stratified data by looking at risk factors specific to premenopausal women and/or postmenopausal women (8–10). Several factors that increase the risk of breast cancer have been identified in postmenopausal women. Data can also be stratified by cancer type which may have different risk factors when modeled independently that vary in magnitude. This study could not conduct such analyses due to the small number of breast cancer cases and the lack of information on breast cancer subtype. To accomplish this, future studies would need information on breast cancer subtype and an ample sample size to maximize the power of detecting a statistically significant comparison.

## Conclusion

Tools to help gauge the risk of breast cancer for American Indian communities are non-existent. The creation of validated models could potentially result in data driven estimates in an easy-to-use format and would be useful for studying which factors increase the risk of breast cancer among American Indian women. Existing data for white women is currently being used to inform specific clinical decisions, plan intervention trials and counsel American Indian women about their risk of the disease because data on American Indian women is limited (11).

Conducting this study was an important first step in gaining a better understanding of the breast cancer risk factors among American Indian women in Belcourt, ND. The results support the need to explore additional potential breast cancer risk factors in this population, such as oral contraceptive use, alcohol drinking, active smoking, obesity, diabetes mellitus, menopausal status, and breastfeeding. Since so little is known about the breast cancer risk profile for American Indian women, future studies are needed for other American Indian tribes. Population-based studies with an ample sample size to allow for stratified analyses while exploring multiple breast cancer risk factors in other geographical regions with a substantial American Indian population are needed.

It is important to have data driven, population-specific breast cancer risk estimates so clinicians are better able to predict breast cancer risk at the individual and population levels. Disparities in breast cancer incidence, mortality and screening among American Indians emphasize the need to better understand the risk factors associated with breast cancer in this population. Once breast cancer risk factors are identified, appropriate interventions can be designed and implemented in order to reduce the breast cancer burden in American Indian communities.

## Data Availability Statement

The original contributions presented in the study are included in the article/Supplementary Material, further inquiries can be directed to the corresponding author/s.

## Author Contributions

MN contributed to the conception, design of the study, and wrote the first draft of the manuscript. MK, LB, and KW wrote sections of the manuscript. All authors contributed to manuscript revision, read, and approved the submitted version.

## Funding

This research was supported in part by the University of Minnesota J. B. Hawley Student Research Award and the National Institute of General Medical Sciences of the National Institutes of Health under Award Number U54GM128729.

## Author Disclaimer

The content is solely the responsibility of the authors and does not necessarily represent the official views of the University of Minnesota, University of North Dakota, or the National Institutes of Health.

## Conflict of Interest

The authors declare that the research was conducted in the absence of any commercial or financial relationships that could be construed as a potential conflict of interest.

## Publisher's Note

All claims expressed in this article are solely those of the authors and do not necessarily represent those of their affiliated organizations, or those of the publisher, the editors and the reviewers. Any product that may be evaluated in this article, or claim that may be made by its manufacturer, is not guaranteed or endorsed by the publisher.
