# Supplementary material for: Exploring Clinical Risk Factors for Breast Cancer Among American Indian Women
Source: Front Public Health. 2022 Jun 17;10:840280. doi: 10.3389/fpubh.2022.840280 (PMC9247609; doi:10.3389/fpubh.2022.840280)
Supplement: Supplementary file 1 [file Table_1.pdf]

APPENDIX

Table 1A. Radiology, electronic and medical chart review - percent complete for cases/controls.

	Cases	Controls
	n %	n %
**Total n=419**	141 (100.0)	278 (100.0)
**Radiology review**	110 (78.0)	278 (100.0)
**Electronic**		
**review**	102 (72.3)	277 (99.6)
**Medical**		
**chart review**	70 (49.6)	141 (50.7)
**No Radiology review**	31 (22.0)	0 (0.0)

Table 2A. Prevalence of maternal characteristics from Gail model based on original and adjusted categories.

Original Categories			Adjusted Categories		
	Cases N (%)	Controls N (%)		Cases N (%)	Controls N (%)
**Age at screening**					
**<50**	43 (30.5)	91 (32.73)	**<56**	69 (48.94)	137 (49.28)
**≥50**	98 (69.5)	187 (67.27)	**≥56**	72 (51.06)	141 (50.72)
**Age at first live birth**					
**<20**	72 (51.06)	139 (50.00)	**<18**	36 (25.53)	56 (20.14)
**20 to 24**	48 (34.04)	106 (38.13)	**18 to 19**	36 (25.53)	83 (29.86)
**25 to 29**	5 (3.55)	14 (5.04)	**20 to 21**	31 (21.99)	76 (27.34)
**≥30**	3 (2.13)	8 (2.88)	**≥22**	25 (17.73)	52 (18.71)
**Nulliparous**	13 (9.22)	11 (3.96)	**Nulliparous**	13 (9.22)	11 (3.96)
**Age at onset of menstruation**					
**≥14**	39 (27.66)	98 (35.25)	**≥14**	39 (27.66)	98 (35.25)
**12 to 13**	79 (56.03)	129 (46.40)	**13**	36 (25.53)	69 (24.82)
**<12**			**12**	43 (30.50)	60 (21.58)
	23 (16.31)	51 (18.35)	**<12**	23 (16.31)	51 (18.35)
**Number of previous benign breast biopsies**					
**0**	111 (78.72)	221 (79.50)	**0**	111 (78.72)	221 (79.50)
**1**	25 (17.73)	51 (18.35)	**≥1**	30 (21.28)	57 (20.25)
**≥2**	5 (3.55)	6 (2.16)			
**Total number of first-degree relatives with breast cancer**					
**0**	104 (73.76)	216 (77.70)	**0**	104 (73.76)	216 (77.70)
**1**	31 (21.99)	53 (19.06)	**≥1**	37 (26.24)	62 (22.30)
**≥2**	6 (4.26)	9 (3.24)			
**BMI, kg/m^2**					
**<25**	15 (10.64)	29 (10.43)	**<25**	15 (10.64)	29 (10.43)
**25-30**	50 (35.46)	92 (33.09)	**25 to 29.99**	39 (27.66)	77 (27.70)
**>30**	76 (53.90)	157 (56.47)	**30 to 32.49**	34 (24.11)	66 (23.74)
			**32.50 to 34.99**	22 (15.60)	29 (10.43)
			**≥35**	31 (21.99)	77 (27.70)
**Parity (# of live births)**					
**≥5**	47 (33.33)	103 (37.05)	**≥5**	47 (33.33)	103 (37.05)
**4**	18 (12.77)	44 (15,83)	**3 to 4**	46 (32.62)	108 (38.85)
**3**	28 (19.86)	64 (23.02)	**1 to 2**	35 (24.82)	56 (20.14)
**2**	26 (18.44)	41 (14.75)	**Nulliparous**	13 (9.22)	11 (3.96)
**1**	9 (6.38)	15 (5.40)			
**Nulliparous**	13 (9.22)	11 (3.96)			
